# Factors influencing pigment production by halophilic bacteria and its effect on brine evaporation rates

**DOI:** 10.1111/1751-7915.13319

**Published:** 2018-10-02

**Authors:** Gloria Andrea Silva‐Castro, Anesu Conrad Moyo, Londiwe Khumalo, Leonardo Joaquim van Zyl, Leslie F. Petrik, Marla Trindade

**Affiliations:** ^1^ Institute of Microbial Biotechnology and Metagenomics (IMBM) Department of Biotechnology University of the Western Cape 7535 Bellville Cape Town South Africa; ^2^ Department of Chemistry University of the Western Cape 7535 Bellville Cape Town South Africa

## Abstract

The disposal of reject brine, a highly concentrated waste by‐product generated by various industrial processes, represents a major economic and environmental challenge. The common practice in dealing with the large amounts of brine generated is to dispose of it in a pond and allow it to evaporate. The rate of evaporation is therefore a key factor in the effectiveness of the management of these ponds. The addition of various dyes has previously been used as a method to increase the evaporation rate. In this study, a biological approach, using pigmented halophilic bacteria (as opposed to chemical dyes), was assessed. Two bacteria, an *Arthrobacter* sp. and a *Planococcus* sp. were selected due to their ability to increase the evaporation of synthetic brine. When using industrial brine, supplementation of the brine with an iron source was required to maintain the pigment production. Under these conditions, the *Planococcus* sp. CP5‐4 produced a carotenoid‐like pigment, which resulted in a 20% increase in the evaporation rate of the brine. Thus, the pigment production capability of halophilic bacteria could potentially be exploited as an effective step in the management of industrial reject brines, analogous to the crystallizer ponds used to mine salt from sea water.

## Introduction

Waste brine streams produced by various industrial processes, for example from power plant pre‐treatment processes such as reverse osmosis (RO) and electro reversal dialysis (EDR), have been considered a lost resource due to the high composition of inorganic contaminants (Baciocchi *et al*., 2009). It is imperative that proper management schemes of these waste streams be devised so as to continue exploitation of natural resources like coal in an environmentally sustainable manner. The 29th Worldwide Desalting Inventory indicated that desalination capacity increased to 3.7 million cubic metres per day globally, compared to 3.2 million m^3^ day^−1^ the previous year during the periods June 2015 to June 2016 (Global Water Intelligence and International Desalination Association, 2016). The need to supply water for municipal and industrial uses is being met with an increase in installed capacity of desalination plants. Industrial brines are complex liquid mixtures of various salts with a composition that depends on the quality of the supplied and generated water, pretreatment techniques and the desalination process used (Giwa *et al*., 2017). Minimization of desalination waste brine through its re‐use or safe re‐entry into the hydrological cycle is a critical part of waste water management. The water treatment plant uses an evaporation pond for disposal of its brine effluent, which is concentrated to a manageable sludge. The process of evaporation, through solar heating, is considered the most effective and economic method for the disposal of brine produced by desalination plants in many arid and semi‐arid regions (Ahmed *et al*., 2000). In South Africa, the use of evaporation ponds for brine disposal is more often used in less arid parts of the country, especially in the Gauteng and Mpumalanga provinces where effective evaporation is more challenging and there is a need to enhance evaporation rates (Petrik, 2015). Therefore, brine management has become a significant area of concern, as the brine is often produced faster than it can evaporate, necessitating the construction of ever larger evaporation ponds and also posing a risk to ground water supplies.

The key factor in the effectiveness of these ponds is the evaporation rate, and the ability to remove water from the brine pond at the same rate or faster than it can be generated. One of the ways to increase the evaporation rate would be to increase the temperature in the evaporation pond. To this end, it is important to retain as much of the incident solar radiation and in that way minimize the heat that is lost due to reflection (Pereira *et al*., 2007). A coloured solution absorbs more solar energy than an uncoloured one resulting in an increase in the temperature of the solution. This lowers the surface tension of the water leading to a higher saturation vapour pressure, and a subsequent increase in the evaporation rate (Ahmed *et al*., 2000b). This can be achieved by using dyes such as methylene blue, congo red, bismarck brown and 2‐naphthol green (Keyes, 1967); however, synthetic dyes are not environmentally friendly (Venil *et al*., 2013). An alternative source of pigment to increase solar absorption could be halophilic microorganisms. These microorganisms, able to tolerate salt concentrations as high as 100 g l^−1^, also produce pigments. Their pigments are responsible for the wide variety of orange to red colours seen in solar saltern ponds and absorb radiant energy in the wavelength range of 300–600 nm, which is thought to cause an increase in the brine temperature and enhanced evaporation rate analogous to chloroplasts in plants and what has been observed for soil crusts (Bhosale and Bernstein, 2005; Couradeau *et al*., 2016; Kume, 2017).

Considering the nature of the brine, a biological approach whereby pigmented halophilic bacteria are used to improve evaporation rates could offer an advantage from a cost and environmental compatibility (biodegradability) perspective (Oren, 2010). The production and application of pigmented bacteria is still a field of research which garners much attention as there are many potential industrial processes which could benefit (Venil *et al*., 2013). Bacterial pigments are preferred compared with chemical dyes because of their reduced environmental impact. Despite their distinct advantages, bacterial pigment production is subject to:(i) tolerance of growth conditions such as pH, temperature, nutrient concentration and (ii) the ability to use a particular carbon or nitrogen source. Here we investigate the effect of pigmented *Arthrobacter* and *Planococcus* species on the evaporation rate of brine wastewater generated by South African coal mines, as a method for improving the evaporation rates and thus the effective disposal of these wastewaters.

## Results

### Bacterial isolation and selection of candidates for evaporation study

A total of six unique bacterial strains were isolated from both the eMalahleni Water Reclamation pond and the Cerebos crystallizer salt ponds based on colony morphology and colour. An initial trial (200 ml scale) was conducted to determine which of these isolates could potentially improve evaporation rates. The effect that pigmented isolates had on the evaporation rate was compared with uninoculated TSB medium prepared with synthetic brine (Table S1) and 200 mg l^−1^of methylene blue dye. The methylene blue concentration was selected taking into account the results of the evaporation rates of synthetic brine (Table S2). Addition of methylene blue to a final concentration between 200 and 300 mg l^−1^ resulted in the most amount of liquid loss during the experiment, these results did not show significant difference (*P* = 0.05) between them, therefore 200 mg l^−1^ concentration was selected to compare with the biological treatment. Inoculation with isolates CP5‐4, EP1 and EP3 had a positive effect on the evaporation rate after 24 h, and all performed better than the 200 mg l^−1^ of methylene blue (Table 1). EP3 inoculation showed a high evaporation rate of 0.085 cm per hour between 24–48 h, while CP5‐4 showed the highest evaporation rate after 48 h with 0.094 cm per hour. These results showed significant difference with respect to the synthetic brine control and 200 ppm methylene blue (LSD test; *P* = 0.05), hence these strains were selected for further study.

**Table 1:** Evaporation rate of the seven isolates in 200 ml synthetic brine during the course of experiments

Isolates	NaCl concentration (%)^a^	Evaporation rate (cm h^−1^)			
		0–24 h	24–48 h	48–56 h	56–72 h
BRINE	–	0.027 ± 0.002	0.028 ± 0.002	0.036 ± 0.002	0.041 ± 0.000
BRINE+TSB	–	0.034 ± 0.003	0.038 ± 0.001	0.068 ± 0.005	0.045 ± 0.001
CP2‐2	10	0.037 ± 0.005	0.041 ± 0.001	0.056 ± 0.004	0.039 ± 0.007
CP5‐4	10	0.039 ± 0.004	0.050 ± 0.001^a^	0.094 ± 0.007^a^	0.005 ± 0.000
CP5‐7	5	0.037 ± 0.003	0.046 ± 0.003	0.045 ± 0.001	0.035 ± 0.001
EP1	10	0.043 ± 0.003	0.054 ± 0.003^a^	0.030 ± 0.019	0.029 ± 0.000
EP2	5	0.044 ± 0.003	0.043 ± 0.002	0.048 ± 0.003	0.040 ± 0.004
EP3	5	0.043 ± 0.002	0.085 ± 0.005^a^	0.002 ± 0.000^a^	0.000 ± 0.000
MB	–	0.035 ± 0.006	0.048 ± 0.002	0.038 ± 0.001	0.038 ± 0.001

Mean ± standard deviation.

a Significant difference in parameters between non‐inoculated synthetic brine (BRINE) and each isolate using Multivariate analysis of variance (MANOVA) with LSD (α = 0.05).

**a**. Salt concentration that isolate grew best in.

**Figure 1:**
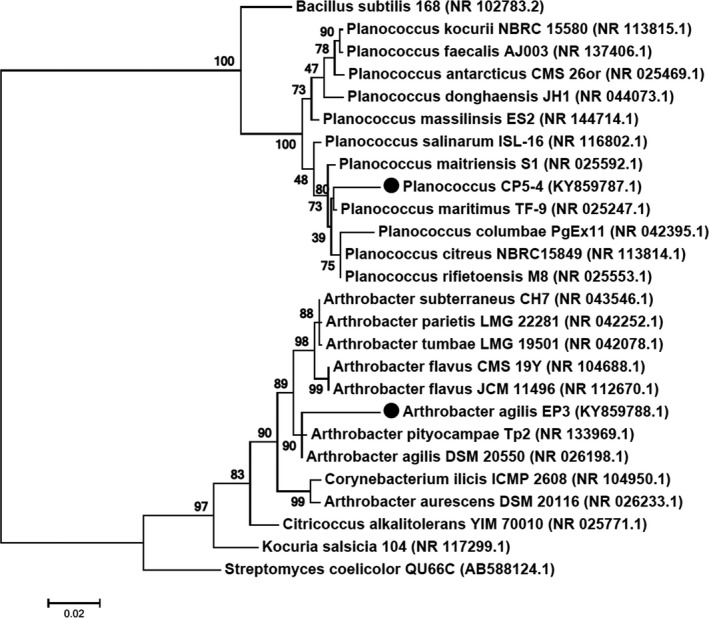
Phylogenetic tree based on 16S rRNA gene sequence of *Arthrobacter* sp. strain EP3 (1402 bp) and *Planococcus* sp. strain CP5‐4 (1423 bp) constructed by Maximum Likelihood method. Numbers on branches indicate bootstrap values after 1000 re‐samplings. Accession numbers of the bacterial species are mentioned in parenthesis. All positions containing gaps and missing data were eliminated. There were a total of 1097 positions in the final dataset.

The 16S rRNA sequence analysis for EP3 and CP5‐4 against the curated NCBI 16S rRNA sequence database indicated that isolate EP3 shared 96% nucleotide identity to *Arthrobacter agilis* (NR_026198.1) and CP5‐4 shared 97% identity with sequence of *Planococcus maritime* (NR_025247.1). The query coverage was 99% and 98% for *A. agilis* and *P. maritimus* using 1402 and 1423 bp sequences respectively. The sequences have been submitted to the GenBank database under accession number KY859788 for strain EP3 and KY859787 for strain CP5‐4. Both these isolates are possibly novel species based on their 16S rRNA sequences and phylogenetic analysis (Fig. 1) which shows a clearly distinct evolutionary relationship of these strains to the members of the respective families.

The salt tolerance of the isolates was tested on R2A broth supplemented with different NaCl concentrations. Isolated strains were able to grow in NaCl concentrations ranging from 0 to 30%. EP3 and CP5‐4 had optimal growth at 5% and 10%, respectively (Table 1), making these moderate halophiles (Ventosa *et al*., 1998).

### Growth and pigment production of EP3 and CP5‐4 isolates in brine (NuW)

**Figure 2:**
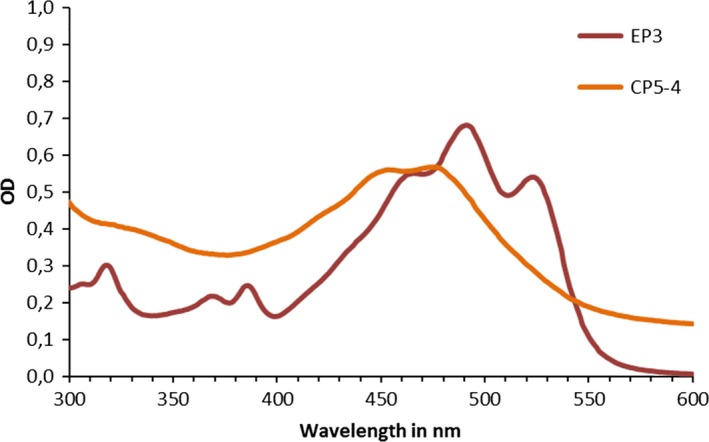
UV/visible scanning spectra of total pigment by EP3 and CP5‐4 isolates from synthetic brine medium.

An initial characterization of the pigment(s) produced by EP3 and CP5‐4 was performed using synthetic brine amended with 100% strength TSB. As demonstrated in Fig. 2, the UV/visible spectrum of the pigment produced by these two organisms illustrated a red‐pink pigment produced by EP3 and orange pigment produced by CP5‐4 with absorption maxima at 495 and 475 nm respectively. The production of these pigments was hypothesized to be the main contributors to the increased evaporation rates observed.

**Figure 3:**
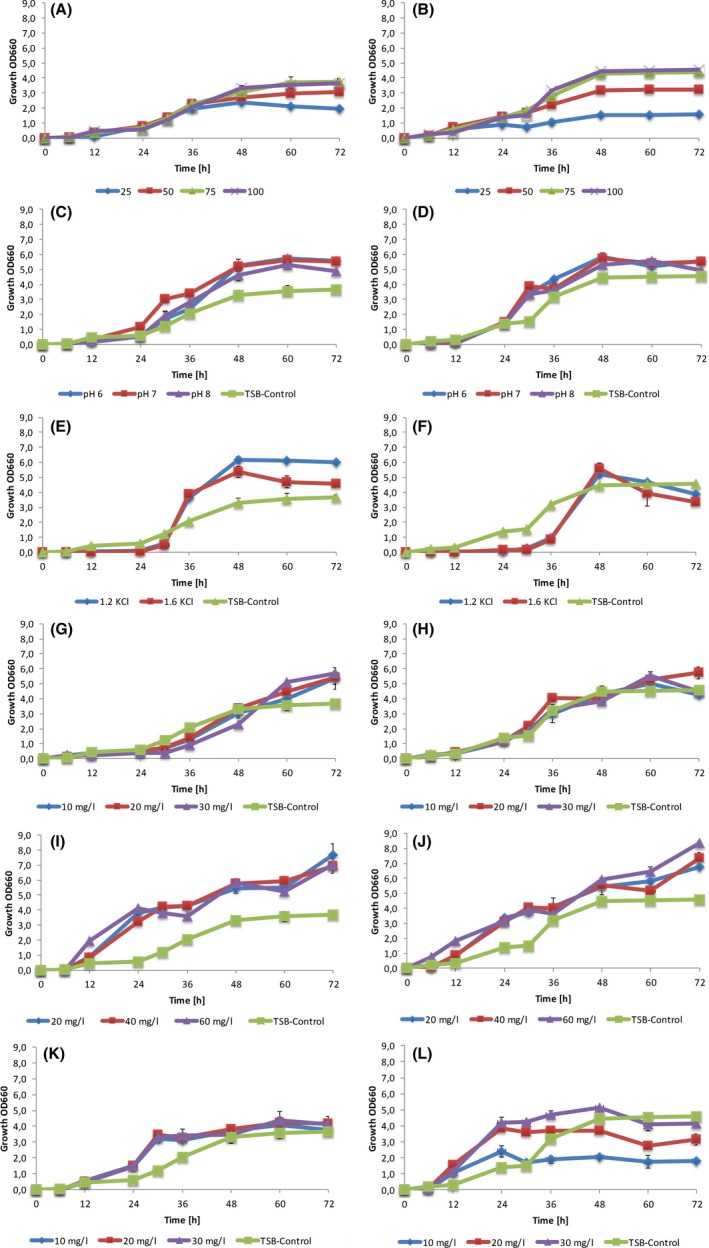
Influence of amount of TSB, pH, KCl, FeCl_3_, FeSO_4_ and NaSO_4_ on growth rate of isolates in brine. A. EP3 in brine with different percentages of TSB; B. CP5‐4 in brine with different percentages of TSB; C. EP3 in brine with pH adjusted to 6, 7 and 8. pH of TSB‐Control ranges was 8.2–8.5; D. CP5‐4 in brine with pH adjusted to 6, 7 and 8. pH of TSB‐Control ranges was 8.2–8.5; E. EP3 in brine with different KCl concentration; F. CP5‐4 in brine with different KCl concentration; G. EP3 in brine with different FeCl_3_ concentration; H. CP5‐4 in brine with different FeCl_3_ concentration; I. EP3 in brine with different FeSO_4_ concentration; J. CP5‐4 in brine with different FeSO_4_ concentration; K. EP3 in brine with different NaSO_4_ concentration; L. CP5‐4 in brine with different NaSO_4_ concentration. Data points represent the average of a minimum of three independent experiments.

To assess the ability of these strains to increase the evaporation rate of industrial brine as opposed to a synthetic version, pigment production by these two strains was assessed in brine supplied by NuWater. The brine did not, however, support high‐level pigment production by the organisms. To establish what the cause of this may be, a chemical analysis of NuWater brine was conducted and compared to that of the brine from eMalahleni (Table S1). The main differences were the levels of chloride, potassium, sulphates and nitrates, which were higher in the eMalahleni brine. To optimize the conditions under which both high cell yields, and pigment production could be achieved, we evaluated the growth and pigment production for the selected isolates in the NuWater brine supplemented with various concentrations of TSB medium, KCL, NaSO_4_, FeCl_3_ and FeSO_4_ under various pH levels (Fig. 3).

When testing the effect of TSB supplementation, the isolates reached a peak growth rate after 48 h for all TSB concentrations (Fig. 3A and B). For both isolates, growth in 75% TSB brine resulted in growth that was comparable to that in 100% strength TSB brine medium. EP3 performed marginally better than CP5‐4 in lower TSB concentrations; however, neither isolate could reach and maintain the optical densities that they could at higher concentrations. Changes in pH did not have an appreciable effect on the growth of CP5‐4 (Fig. 3D). KCl addition for EP3 resulted in the stimulation of growth after 24 h of incubation (Fig. 3E), although CP5‐4 did not show a significant difference compared with non‐supplemented medium (Fig. 3F). With the addition of iron to full strength TSB‐brine medium, the isolates experienced rapid growth rates after 12 h of incubation in all FeCl_3_/FeSO_4_ concentrations evaluated. Addition of iron also resulted in both strains having a prolonged exponential growth phase and both isolates grew to higher final optical densities (Fig. 3G–J). Figure 3K and L show that the addition of sulphate stimulates growth after 12 h of incubation.

**Figure 4:**
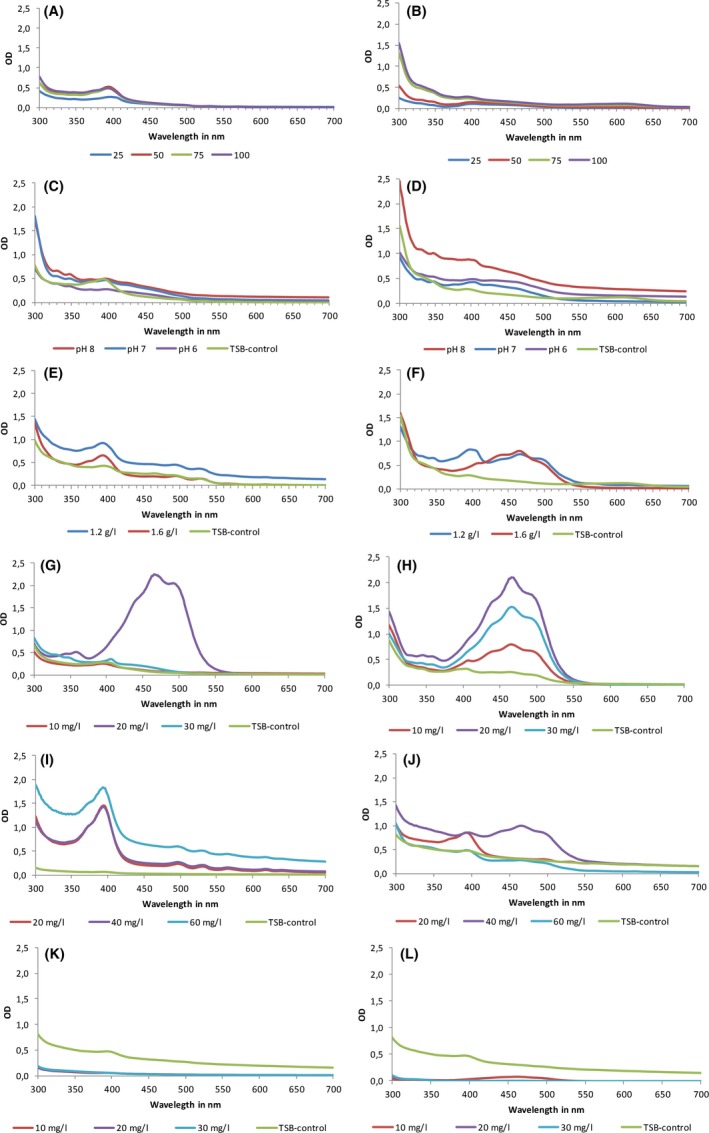
Absorption spectra of the carotenoids extracted from EP3 and CP5‐4 cultures with different concentrations of TSB, pH, KCl, FeCl_3_, FeSO_4_ and NaSO_4_ in brine at 48 h of incubation. A. EP3 in brine with 25%, 50%, 75% and 100% of TSB; B. CP5‐4 in brine with 25%, 50%, 75% and 100% of TSB; C. EP3 in brine with pH adjusted to 6, 7 and 8, pH of TSB‐Control ranges was 8.2–8.5; D. CP5‐4 in brine with pH adjusted to 6, 7 and 8, pH of TSB‐Control ranges was 8.2–8.5; E. EP3 in brine with different KCl concentration; F. CP5‐4 in brine with different KCl concentration. G. EP3 in brine with different FeCl_3_ concentration; H. CP5‐4 in brine with different FeCl_3_ concentration; I. EP3 in brine with different FeSO_4_ concentration; J. CP5‐4 in brine with different FeSO_4_ concentration; K. EP3 in brine with different NaSO_4_ concentration; L. CP5‐4 in brine with different NaSO_4_ concentration. Data points represent the average of a minimum of three independent experiments.

Pigment production of EP3 and CP5‐4 was also evaluated under these growth conditions (Fig. 4).

The concentrations of TSB‐brine, NaSO4 and pH did not appear to affect pigment production, unlike what was observed for cell growth. Increasing the amount of potassium and chloride in the full‐strength TSB‐brine medium stimulated mainly orange pigment production in CP5‐4 (Fig. 4F). Addition of KCl to 1.6 g l^−1^ resulted in pigment with an absorption maximum at ±460 nm and a yield of 0.860 Abs_460_/gDCW/ml, whereas addition of KCl to 1.2 g l^−1^ resulted in a change in the absorbance spectrum with a new peak appearing at 400 nm leading to a decreased yield of orange pigment at 0.512 Abs_460_/gDCW/ml (Table 2). EP3 extracts showed peak absorbance at a wavelength of 395 nm while the 495 and 530 nm wavelength peaks were substantially reduced (Fig. 4E). The yield of the pigment produced by isolate EP3 was 0.34 Abs_495_/gDCW/ml with addition of 1.2 g l^−1^ KCl and 0.39 Abs_495_/dGCW/ml with 1.6 g l^−1^ of KCl (Table 2).

**Table 2:** Yield and efficiency of the pigment production by the isolates Ep3 and CP5‐4 during the course of the evaporation rate experiments

Condition	Time (h)^a^	λ_Max_ (nm)^a^	Yield (Abs/gDCW/ml)	Efficiency
EP3				
Full strength TSB	24	400	0.71 ± 0.13	0.53 ± 0.13
pH 6	72	400	0.44 ± 0.03	0.24 ± 0.04
KCl				
1.2 g l^−1^	48	495	0.34 ± 0.07	0.19 ± 0.04
1.6 g l^−1^	48	495	0.39 ± 0.08	0.22 ± 0.05
FeCl_3_				
10 mg l^−1^	48	400	0.60 ± 0.02	0.35 ± 0.10
20 mg l^−1^	48	495	3.29 ± 1.13	2.31 ± 0.11
30 mg l^−1^	24	400	0.93 ± 0.27	0.59 ± 0.17
FeSO_4_				
20 mg l^−1^	48	495	0.25 ± 0.02	0.12 ± 0.01
40 mg l^−1^	48	495	0.61 ± 0.01	0.30 ± 0.02
60 mg l^−1^	48	495	0.61 ± 0.03	0.30 ± 0.02
CP5‐4				
Full strength TSB	72	400	0.17 ± 0.05	0.08 ± 0.02
pH 8	48	400	0.49 ± 0.04	0.29 ± 0.03
KCl				
1.2 g l^−1^	72	460	0.86 ± 0.26	0.52 ± 0.19
1.6 g l^−1^	24	460	0.51 ± 0.14	0.37 ± 0.06
FeCl_3_				
10 mg l^−1^	24	460	4.75 ± 1.40	4.53 ± 1.35
20 mg l^−1^	24	460	4.44 ± 0.64	3.40 ± 1.55
30 mg l^−1^	24	460	3.23 ± 1.06	2.59 ± 0.64
FeSO_4_				
20 mg l^−1^	72	460	0.70 ± 0.12	0.37 ± 0.03
40 mg l^−1^	72	460	0.52 ± 0.01	0.10 ± 0.02
60 mg l^−1^	72	460	0.16 ± 0.03	0.081 ± 0.01

**a**. Time (h) and wavelength (nm) at which the highest pigment OD was obtained during the course of the experiments.

Significant changes in pigment production were observed when TSB‐brine was supplemented with iron after 24 h of incubation. Addition of FeCl_3_ increased the production of the orange pigment by CP5‐4 as well as the red‐pink pigment by EP3. In the case of CP5‐4, there was an increase in the yield of orange pigment with increased FeCl_3_ concentrations, with yields of 4.4 and 4.75 Abs_460_/gDCW/ml at 10 and 20 mg l^−1^ FeCl_3_ respectively (Table 2). However, at 30 mg l^−1^ a decrease was observed. In the case of EP3, addition of 20 mg l^−1^ FeCl_3_ improved pigment yield to 3.29 Abs_495_/gDCW/ml, however, high concentrations of FeCl_3_ did not stimulate production of the red‐pink pigment.

With addition of FeSO_4_, EP3 extracts showed highest absorption at 400 nm as well as two smaller peaks at 495 nm and 525 nm, suggesting the presence of a small amount of the red pigment (Fig. 4I). The CP5‐4 methanol extract absorbed maximally at 460 nm after addition of 40 mg ml^−1^ of FeSO_4_, which was indicative of the presence of the orange pigment (Fig. 4J), and the yield of pigment produced was 0.52 Abs_465_/gDCW/ml under these conditions (Table 2).

### Evaporation rate studies

**Figure 5:**
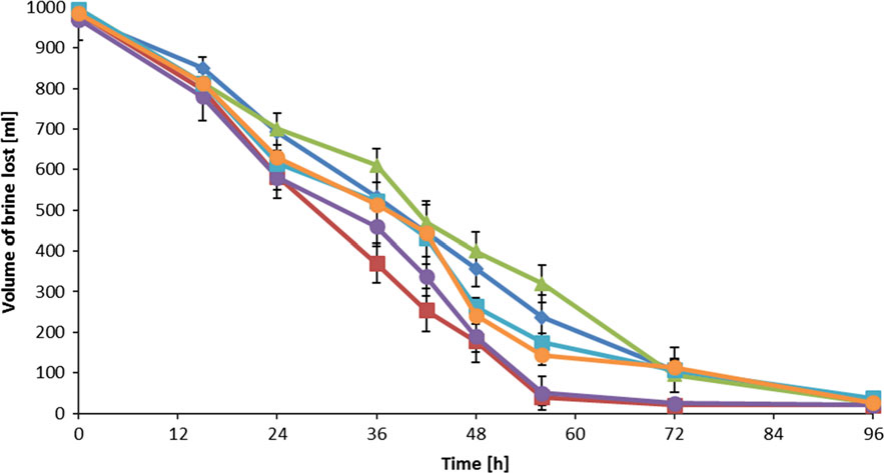
Brine loss over time after inoculation by CP5‐4 on brine supplied with FeSO_4_ and FeCl_3_. (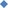)Iron chloride control (Brine+ 20 mg l^−1^ of FeCl_3_); (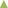) Iron sulphate control (Brine + 40 mg l^−1^ of FeSO_4_); (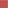)10% v/v of CP5‐4 bacteria inoculated on brine supplemented with FeCl_3_ (10% CP5‐4 in NuWater + FeCl_3_); (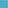) spent TSB medium in brine supplemented with FeCl_3_ (10% spent TSB in NuWater + FeCl_3_); (●)10% v/v of CP5‐4 bacteria inoculation on brine supplemented with FeSO_4_ (10% CP5‐4 on NuWater + FeCl_3_). (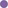)spent TSB medium in brine supplemented with FeSO_4_ (10% spent TSB in NuWater + FeSO_4_).

CP5‐4 was selected to perform evaporation rate studies in NuW brine due to its superior pigment producing ability in this brine supplemented with FeCl_3_ and FeSO_4_. Culturing the isolate in the NuW brine supplied with iron resulted in an increased evaporation rate over the first 12 h compared to the uninoculated controls (Fig. 5).

**Figure 6:**
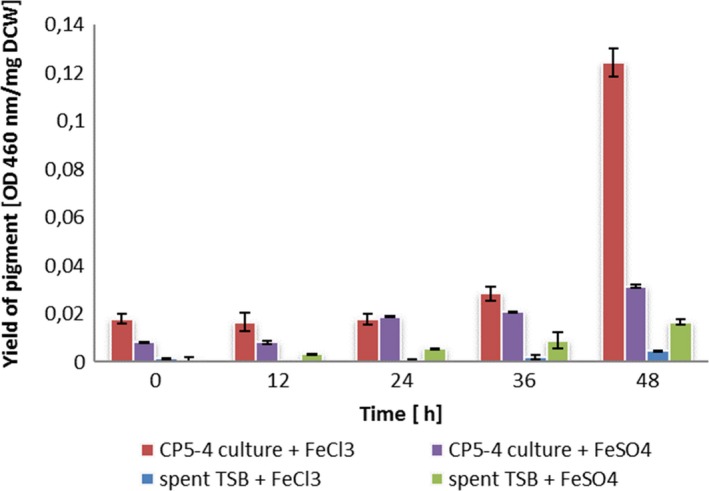
Yield of CP5‐4 pigment, evaporation rate as a function of time. Yield is expressed in pigment OD per dry weight of cell. (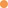), CP5‐4 bacteria inoculated on brine supplemented with FeCl_3_ (CP5‐4 culture + FeCl_3_); (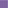), spent TSB medium in brine supplemented with FeCl_3_ (spent TSB + FeCl_3_); (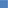), CP5‐4 bacteria inoculation on brine supplemented with FeSO_4_ (CP5‐4 culture + FeCl_3_). (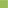), spent TSB medium in brine supplemented with FeSO_4_ (spent TSB+ FeSO_4_).

There appeared to be no difference in the CP5‐4 evaporation rate between FeSO_4_ and FeCl_3_ supplemented brine with values of 0.0325 ± 0.003 and 0.0324 ± 0.003 cm per hour respectively. An increase in the evaporation rate of between 20% and 30% when inoculated with CP5‐4 was observed. High pigment yield after 48 h was achieved with the CP5‐4 inoculation when supplemented with FeCl_3_ (Fig. 6), where the yield reached a value of 0.12 OD_460_ per mg of DCW.

In the case of FeSO_4_, CP5‐4 gradually produced pigment until OD_460_ 0.031 per mg DCW after 48 h. It is noteworthy that the increase in evaporation rate was concomitant with pigment production, suggesting that the bacterial pigment was the major contributor to the increased evaporation rate.

## Discussion

Microbial pigmentation has been studied for centuries (Engelmann, 1882), and with the increased awareness in human safety and environmental conservation, a trend towards their application as eco‐friendly and biodegradable commodities has followed from this emerging field of study (Venil and Lakshmanaperumalsamy, 2009). Pigments are mostly employed as colouring agents in the pharmaceutical, cosmetic, textile and food industries. In the crystallizer brines of saltern ponds, for the production of salt from seawater, halophilic microorganisms are well known for being responsible for the red colour of the brines at or approaching salt saturation. Different types of pigments, ranging from red to purple, contribute to the coloration of the brines. It has been proposed that in these systems, the pigments increase solar energy absorbance by the brine thus resulting in an increased evaporation rate (Zhiling and Guangyu, 2009).

The purpose of this study was to establish whether pigment‐producing halotolerant bacteria could be used as a biological treatment of industrial brines through increased evaporation. Although the influence on evaporation by pigmented microorganisms in salterns has been postulated, here we present for the first‐time evidence that addition of pigmented isolates to industrial brine increases the evaporation rate.

Carotenoids are produced by many phylogenetically distinct non‐photosynthetic bacterial groups. The *Arthrobacter* genus, widely distributed throughout various environments, is well known for its ability to produce a great variety of pigment hues and rather uncommon structures (Sutthiwong *et al*., 2014). The red‐pink pigment produced by the *Arthrobacter* sp. EP3 could tentatively be identified as a derivative of bacterioruberin. Data reported in the literature provide support that bacterioruberin is a characteristic carotenoid from halophilic microorganisms (Abbes *et al*., 2013), and the spectral peaks characteristic of red carotenoid exhibit maximal absorption at 467, 493 and 527 nm (Britton, 1995), similar to what we observed in this study. The main type of carotenoid produced by *Planococcus* species has been described as Glyco‐C30‐carotenoic acid. These terpenoids possess a chain of 30, 40 or 50 carbons, with absorption peaks at 450–490 nm (Kim *et al*., 2015; Ganapathy *et al*., 2016). The *Planococcus* sp. CP5‐4 pigment had a similar absorption spectrum to that described and could therefore tentatively be assigned as a C30‐carotenoic acid.

Generally, carotenoid production occurs in response to environmental conditions such as growth temperature, light and salt concentration, and the investigation of their regulatory mechanisms has provided insight into the adaptation of bacteria to their respective environment (Sutthiwong *et al*., 2014). For example, red carotenoids have been proposed to increase the resistance of heterotrophic bacteria to environmental stress as being cryo‐ and solar radiation protectants (Dieser *et al*., 2010).

As mentioned before, biological approaches are premised on the application of a dye/pigment to brine to trap more solar radiation in a wavelength range and the absorption of this light/heat energy will be transferred to the surrounding water body, thereby raising its temperature. Our results showed a 21% increased evaporation rate was achieved following addition of methylene blue dye at a concentration of 200 ppm, whereas the highest increase (51%) was achieved with the biological approach. Methylene blue has two absorbance wavelength ranges (200–350 and 550–700 nm), whereas the pigments produced by halophilic bacteria often absorb maximally in the wavelength range (400–600 nm). This happens (through evolution) to correspond to the wavelength range of maximum solar energy output and, depending on what they are dissolved in, have a molar extinction coefficient that matches or exceeds that of methylene blue: Astaxanthin – 125 000 in DMSO; bacterioruberin – 141 000 in acetone; methylene blue – 70 000 in water. Therefore astaxanthin and bacterioruberin should perform as well or better at absorbing and transferring solar energy (electronic excitation converted to energy of motion in the atoms of the pigment) to a body of water than dyes such as methylene blue. This is why the pigment produced by CP5‐4 and EP3 perform as well or better at absorbing and transferring solar energy compared to methylene blue (Harbeck, 1955; Moore and Runkles, 1968).

In this study, the EP3 and CP5‐4 pigment production was not constitutive, where pigmentation was absent when cultured in the NuWater brine, whereas pigmentation was observed in the synthetic brine supplemented with TSB. Schobert and Jahn (2002) proposed that changes in environmental conditions could lead to this as a result of an adaptation of the energy conserving electron transport chain and cofactors of various enzymes and thus causing significant changes in pigment production (Sutthiwong *et al*., 2014). The biological roles of ions such as potassium, magnesium, sodium, calcium and other transition elements like metal ions in microbes are difficult to study because they interact weakly with carrier ligands (Bhosale, 2004). However, supplementation of inorganic salts to the culture medium was reported to affect or stimulate carotenogenes in *Haematococcus pluvialis* and *Rhodotorula*.

The pigment production behaviour observed in ion supplemented medium was different for each isolate. Addition of iron in the form of FeCl_3_ and FeSO_4_ promoted pigment production for isolate CP5‐4, which led to increased evaporation rates, however the same effect was not observed for EP3, which showed decreased pigment production under the conditions tested. Although we did not observe accumulation of the bacterioruberin‐like pigment in EP3 after addition of FeSO_4_, we did observe the production of a second pigment (maximum absorbance at ~390 nm; Fig. 6) suggesting that a different pigment production pathway was induced. In CP5‐4, we observed reduced bacterioruberin‐like pigment production as well as a shift in the wavelength scan profile of the pigments produced, when the bacterium was grown on FeSO_4_. Thus, both bacteria still produce pigments when cultured with FeSO_4_; however, the production is elevated when supplementing with FeCl_3_. Iron appears to play a role in pigment production, and it is possible that the various forms of iron (ferrous vs. ferric) could have a direct and different regulatory effect on the genes involved with pigment production. On the other hand, both bacteria also grew better in response to the addition of FeSO_4_ as opposed to FeCl_3_ (Fig. 5). The general growth state of the cells determines whether pigment is produced or not (serves to regulate pigment production), and the difficulty in acquiring biologically available (ferrous) iron from FeCl_3_ induces secondary metabolism due to strained growth. The induction of secondary metabolite pathways as a result of inducing stress is a well‐known phenomenon.

Several studies suggest that hyper‐accumulation of astaxanthin by *Haematococcus pluvialis* induced by ferrous iron was due to the generation of hydroxyl radicals from the Fenton reaction, which stimulates cellular carotenoid synthesis (Kobayashi *et al*., 1992; Tjahjono *et al*., 1994; Bhosale, 2004). In the same way, Bhosale and Gadre (2001) reported that *Rhodotorula* showed a marked improvement in the production of carotenoids due to a stimulatory effect of copper, zinc and ferrous iron on carotenoid‐synthesizing enzymes or to the generation of oxygen radicals in the culture broth. In this regard, these metals have the same effect as ionizing radiation on the cultures. The observation that each isolate produced two possible pigments with different absorbance spectra as well as different responses to the addition of iron could point to different modes of regulation and/or biosynthesis.

The main results of this study concluded that the pigment produced by CP5‐4 was sufficient to increase brine evaporation rates in a controlled system. We therefore demonstrate for the first time the ability of pigmented, halotolerant bacteria to increase evaporation rates in brine ponds and foresee that this may become a viable option to improve the throughput in these ponds. Whether the same effect can be extrapolated to an evaporation pond system has yet to be tested, since many factors that directly contribute to evaporation, such as fluctuating temperatures, wind, varying pond depth, were not assessed in this study. Another critical factor to consider in future studies is the adaptability of the CP5‐4 strain to the evaporation pond conditions. For this approach to be feasible, the strain would have to proliferate and maintain pigment production under changing conditions, as would be experienced in an evaporation pond including a reduced need for additional supplementation with nutrients. However, a number of genetic engineering options could be considered towards designing improved performance, for example, by promoter refactoring for constitutive expression of the carotenoid pathway.

## Experimental procedures

### Brine samples

Brine samples used in this study were collected from three different evaporation ponds; the eMalahleni Water Reclamation pond situated in the Mpumalanga province, South Africa (S 25°56′41.4, E 29°11′67.0); the Cerebos crystallizer salt ponds in Velddrif, Western Cape, South Africa (S 32°47′10,632, E 18°10′9,499); and NuWater Global, Epping, South Africa. The composition of the eMalahleni and NuWater brines were determined by Bemlab (South Africa) (Table S1). A synthetic brine was formulated based on the composition of the eMalahleni brine, with the following composition (w/v): 3497 mg l^−1^ Na^+^; 758 mg l^−1^ K^+^; 223 mg l^−1^ Mg^2+^; 964 mg l^−1^ Ca^2+^; 762 mg l^−1^ Cl^‐^ and 10 255 mg l^−1^ ${\text{SO}}_{4}^{2-}$.

### Microorganisms and culture conditions

Bacterial strains employed in this study (EP3 and CP5‐4) were selected from six strains isolated from the eMalahleni and Cerebos brine samples. To isolate bacteria from the eMalahleni brine, 1l of brine was vacuum filtered through a 0.22 μm membrane filter. The membrane was washed, and the cells were suspended in 1 ml of sterilized eMalahleni brine. One gram of each of the Cerebos samples were suspended in 10 ml of sterile synthetic brine and mixed by vortexing. A serial dilution was prepared from the bacterial suspensions and inoculated by spreading onto TSB agar plates prepared using sterile eMalahleni brine instead of dH_2_O. Pigmented isolates growing on the plates were picked and streaked on TSB‐brine agar (17 g l^−1^ Pancreatic digest of casein; 3 g l^−1^ Enzymatic digest of soya bean; 5 g l^−1^ Sodium chloride; 2.5 g l^−1^ Dipotassium hydrogen phosphate; 2.5 g l^−1^ Glucose; 14 g l^−1^ Agar). The growth of the isolates was also evaluated over a range of NaCl concentrations (0.5 to 30% w/v) in R2A medium liquid (Yeast extract 0.50 g; Proteose Peptone 0.50 g; Casamino acids 0.50 g; Glucose 0.50 g; Soluble starch 0.50 g; Na‐pyruvate 0.30 g; K_2_HPO_4_ 0.30 g; MgSO_4_ × 7 H_2_O 0.05 g; 1L of Distilled water).

The strains were identified by analyzing the 16S rRNA gene sequence. Genomic DNA was extracted from bacteria cultured in 15 ml of R2A broth supplemented with 5% of NaCl with shaking at 120 rpm at room temperature, and genomic DNA extraction was performed using the method described by Martín‐Platero *et al*. (2007). PCR amplification was performed in 50 μl volumes which contained 100 ng template DNA, 1.25 units of NEB Taq polymerase, 10× NEB PCR buffer, 0.2 mM of deoxynucleoside triphosphates (dNTPS) and 0.1 μM of each primer. The universal primer set E9F (Hansen *et al*., 1998) and U1510R (Reysenbach and Pace, 1995) was used for amplification. The following thermos cycling conditions were used: initial denaturation at 95°C for 4 min (1 cycle); 30 cycles of denaturation at 95°C for 30 sec, annealing at 55°C for 30 s, extension at 72°C for 90 s and final extension at 72°C for 7 min. After gel purification, using a Nucleospin kit (Machery‐Nagel), Sanger sequencing of 16S rRNA gene fragments was carried out by the Central Analytical Facility at Stellenbosch University using an ABI PRISM 377 automated sequencer and the sequence compared with the NCBInr database using BLASTn to identify the isolates. The maximum likelihood phylogenetic tree was constructed using MEGA 7 software (Kumar *et al*., 2016).

### Optimization of parameters for pigment production

The optimization of pigment production was performed using NuWater brine (Table S1). Initially, bacterial growth was assessed in TSB‐brine (NuW) medium with varying percentages of TSB from 25% to 100% TSB w/v, 100 μl of an overnight culture was inoculated in 50 ml of medium and incubated at room temperature, with growth measured by OD Reading at 660 nm every 12 h for a period of 3 days. Inoculums for growth rate and pigment production were prepared by diluting the culture to an OD600 nm of 1. The effect of the following parameters on pigment production was assessed in 50 ml cultures in Erlenmeyer flasks, which were independently assessed: pH, [KCl], [FeCl_3_], [FeSO_4_], [NaSO_4_]. One hundred microlitres of EP3 and CP5‐4 overnight culture was inoculated into fresh full‐strength (100% w/v) TSB‐brine (NuW) with or without supplementation. To determine the effect of pH on pigment production, the pH of the media was adjusted to pH values 6, 7 and 8. The effect of KCl was determined using concentrations 1.2 and 1.8 g l^−1^. Ferric chloride (FeCl_3_) and ferrous sulphate (FeSO_4_) were used to study the effect of iron on pigment production with concentrations ranging between 0.035–12 mg l^−1^ (Rachid and Ahmed, 2005). Sodium sulphate (NaSO_4_) was used to determine the effect of sulphate, using 6–40 mg l^−1^. The lowest concentrations were selected to match the eMalahleni brine concentration in all cases. Growth was determined spectrophotometrically at 660 nm. One millilitre of the culture was used to measure pigment production as well as increase in cell biomass, by pipetting into pre‐weighed Eppendorf tubes. The cells were collected by centrifugation at 7274 *g* for 10 min. The supernatant was removed, and the pellet washed with PBS and air‐dried before determining the cell biomass. Cells were resuspended in 1 ml of methanol and incubated for 15 min at 60⁰C, after which the cell suspension was centrifuged for 10 min at 10 000 RCF and the methanol extract (supernatant) used for spectrophotometric analyses. To determine absorbance maxima, UV/visible scanning spectra of extracts were recorded between 200 and 800 nm. The pigment yield in the methanol extract was calculated using the formula (1) (Fang *et al*., 2010).

(1) $$\begin{array}{cc} \text{yield} & =\frac{\text{Absorbance maxima of methanol extract (Abs)}}{\text{dry weight biomass (g)}}\\ & \times \text{volume of culture (ml)} \end{array}$$

### Evaporation rate measurements

The effect of isolates on evaporation rates was assessed at two different scales: (i) initially in glass Petri‐style dishes with 200 ml of synthetic brine inoculated with an overnight culture of the six isolates as well as 200 mg l^−1^ of methylene blue dry (Table 1) and (ii) in a glass pan that was 27 cm in length, 20 cm in width and 7.5 cm in height (Fig. S1), with 900 ml of brine (NuW) inoculated with EP3 and CP5‐4 cultures grown in 100 ml of TSB‐brine (NuW) to reach a final volume of 1l in the pan. In both scenarios the brine was evaporated using 240‐Watt infrared lamps situated approximately 40 cm above the surface of the cultures as a source of heat. The overnight cultures were standardized to an optical density of 1 at 660 mn.

The amount of brine lost was measured at 6‐h intervals, until the pan was completely dry. One millilitre of sample was also collected at each interval to measure pigment production and growth. The rate of evaporation of the brine was calculated as in (2) (Ladewig and Asquith, 2011):

(2) $$\begin{array}{c} \text{Evaporarion rate}\;(\frac{\text{cm}}{h})\\ =\frac{\text{volume of the brine lost over time}\;(\frac{{\text{cm}}^{3}}{h})}{\text{Surface area}\;\left({\text{cm}}^{2}\right)} \end{array}$$

In the 1 l experiment, six growth conditions were assessed for each culture :(i) Brine (NuW) + FeCl_3_; (ii) Brine (NuW) + FeSO_4_; (iii) spent TSB‐brine (NuW) + FeCl_3_; (iv) spent TSB‐brine (NuW) + FeSO_4_; (v) Brine (NuW) + FeCl_3_+EP3/CP5‐4 inoculum; (vi) brine (NuW) + FeSO_4_+EP3/CP5‐4 inoculum; spent TSB consisted of the supernatant of an overnight culture in TSB‐brine medium of each isolate, cleared first through centrifugation at 5520 *g* for 10 min then filtered through a 0.22 μm membrane filter.

### Statistical analysis

Each treatment was conducted in triplicate to calculate the mean values and respective standard deviations. Multivariate analysis of variance (MANOVA) with LSD test was performed to determine the statistically significant differences. All statistical analyses were carried out using the SPSS (Statistics for Windows, Version 15.0. Armonk, NY: IBM Corp).

## Conflicts of interest

None declared.

## Supporting information


**Fig. S1.** Set‐up of evaporation rate assays.

**Table S1.** Chemical and physical properties of the brine samples.

**Table S2.** Average evaporation rates of synthetic brine with various concentrations of methylene blue dye in 200 ml synthetic brine.
