# Supplementary figures and images for: Factors influencing pigment production by halophilic bacteria and its effect on brine evaporation rates
Source: Microb Biotechnol. 2018 Oct 2;12(2):334–45. doi: 10.1111/1751-7915.13319 (PMC6389849; doi:10.1111/1751-7915.13319)

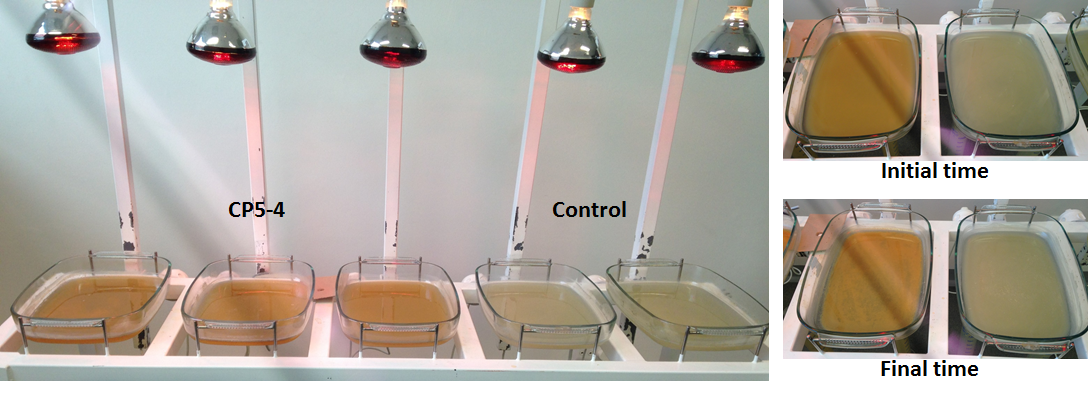

Supplement: Supplementary file 1 — Fig. S1. Set‐up of evaporation rate assays. [file MBT2-12-334-s001.tif]
