# Supplementary material for: Factors influencing pigment production by halophilic bacteria and its effect on brine evaporation rates
Source: Microb Biotechnol. 2018 Oct 2;12(2):334–45. doi: 10.1111/1751-7915.13319 (PMC6389849; doi:10.1111/1751-7915.13319)
Supplement: Supplementary file 2 — Table S1. Chemical and physical properties of the brine samples. [file MBT2-12-334-s002.docx]

**Table S1.**Chemical and physical properties of the brine samples.

|  | **eMalahleni** | **NuWater** |
| --- | --- | --- |
| **pH – Value at 25°C** | 7.76 | 8.2 |
| **Electrical Conductivity in**  **mS/m at 25°C** | 2190 | 1780 |
| **Chloride** | **1072.78** | 539 |
| **Sulphate** | **14520** | 10871 |
| **Fluoride** | ND | 2.9 |
| **Nitrate** | 122.12 | 0.1 |
| **Nitrite** | 0.01 | 0.4 |
| **Sodium** | 3973 | 3859 |
| **Potassium** | **710** | 104 |
| **Calcium** | 1200 | 985 |
| **Magnesium** | 459 | 837 |
| **Aluminium** | 0.03 | <0.10 |
| **Barium** | 0.21 | 0.172 |
| **Copper** | 0.004 | <0.025 |
| **Iron** | **0.07** | 0.043 |
| **Manganese** | 0.01 | 0.422 |
| **Phosphorus** | 0.03 | 1.64 |
| **Zinc** | 0.008 | 0.571 |

Concentration of the components of the both brines is expressed in ppm
