# Supplementary material for: Factors influencing pigment production by halophilic bacteria and its effect on brine evaporation rates
Source: Microb Biotechnol. 2018 Oct 2;12(2):334–45. doi: 10.1111/1751-7915.13319 (PMC6389849; doi:10.1111/1751-7915.13319)
Supplement: Supplementary file 3 — Table S2. Average evaporation rates of synthetic brine with various concentrations of methylene blue dye in 200 ml synthetic brine. [file MBT2-12-334-s003.docx]

**Table S2:** Average evaporation rates of synthetic brine with various concentrations of methylene blue dye in 200 mL synthetic brine.

| **Methylene blue**  **concentration**  **[mg·L^-1^]** | **Evaporation rate**  **[cm/ h]** |
| --- | --- |
| 0 | 0.027 ± 0.002 |
| 100 | 0.034 ± 0.003 |
| 150 | 0.037 ± 0.005 |
| 200 | 0.039 ± 0.004 |
| 250 | 0.037 ± 0.003 |
| 300 | 0.043 ± 0.003 |

Mean ± standard deviation
